# Optical and Photoacoustic Properties of Laser-Ablated Silver Nanoparticles in a Carbon Dots Solution

**DOI:** 10.3390/molecules25245798

**Published:** 2020-12-09

**Authors:** Amir Reza Sadrolhosseini, Ganesan Krishnan, Suhaidi Shafie, Suraya Abdul Rashid, Sulaiman Wadi Harun

**Affiliations:** 1 Functional Device Laboratory, Institute of Advanced Technology, Universiti Putra Malaysia, Seri Kembangan 43400, Malaysia; 2 Laser Center, IBNU Sina Institute for Scientific and Industrial Research, Universiti Teknologi Malaysia, Johor Bahru 81310, Malaysia; k.ganesan@utm.my; 3 Department of Electrical Engineering, Faculty of Engineering, University of Malaya, Kuala Lumpur 50603, Malaysia; swharun@um.edu.my; 4 Material Processing Laboratory, Institute of Advanced Technology, Universiti Putra Malaysia, Seri Kembangan 43400, Malaysia; suraya_ar@upm.edu.my

**Keywords:** silver nanoparticles, z-scan, photoacoustic, laser ablation, fluoride, carbon dots

## Abstract

This study used the carbon dots solution for the laser ablation technique to fabricate silver nanoparticles. The ablation time range was from 5 min to 20 min. Analytical methods, including Fourier transform infrared spectroscopy (FTIR), UV-visible spectroscopy, transmission electron microscopy, and Raman spectroscopy were used to categorize the prepared samples. The UV-visible and z-scan techniques provided optical parameters such as linear and nonlinear refractive indices in the range of 1.56759 to 1.81288 and 7.3769 × 10^−10^ cm^2^ W^−1^ to 9.5269 × 10^−10^ cm^2^ W^−1^ and the nonlinear susceptibility was measured in the range of 5.46 × 10^−8^ to 6.97 × 10^−8^ esu. The thermal effusivity of prepared samples, which were measured using the photoacoustic technique, were in the range of 0.0941 W s^1/2^ cm^−2^ K^−1^ to 0.8491 W s^1/2^ cm^−2^ K^−1^. The interaction of the prepared sample with fluoride was investigated using a Raman spectrometer. Consequently, the intensity of the Raman signal decreased with the increasing concentration of fluoride, and the detection limit is about 0.1 ppm.

## 1. Introduction

Carbon dots (CD) are the new class of nanomaterial. CD is a 0-dimension nanoparticle that has a quasi-spherical carbon nanostructure with a particle size less than 10 nm [1] and the carbon atom contains sp^2^ bonding in the CD structure [2,3]. CD has promising properties such as nontoxic, biocompatible, small size, strong solubility [4] in water and other solvents such as alcohol, robust chemical inertness, high luminescence properties [4,5], facile modification, and photobleaching resistance [6,7]. CD can be used for industry and medicine applications, including bioimage [8], biosensor and sensor [9], photocatalyst application [10], and optoelectronic devices [2] such as solar cell chip.

The metal nanoparticles such as silver (Ag–NPs) and gold nanoparticles (Au–NPs) have unique properties. Therefore, they can be used in medicine, catalysis, optics, microelectronics, medical imaging, environmental remediation, and biosensor [11,12]. Ag–NPs have the surface plasmon resonance peak at the UV–vis range and the surface-plasmon propagation has a minor loss of optical frequency [13]. It is non-toxic, has great electrical and thermal conductivity, high-primitive features, wide absorption of the visible and remote IR region of the light, surface-enhanced Raman scattering, and non-linear optical behavior. According to literature, Ag–NPs can improve the fluorescence properties, surface Raman scattering, Raman properties, and antibacterial effect of carbon-based dots [14,15]. Ag–NPs/CD composite was used to detect H_2_S_2_ [16], silver in aqueous medium [17], sulfide ions [18], and metal ion (Hg^2+^, As^3+^, Pb^2+,^ Cd^2+^, and Cu^2+^) [19]. Therefore, Ag–NPs/CD has the potential for sensing and degrading toxic chemicals. 

The laser ablation technique is a unique, fast and simple method to fabricate the metal and metal oxide nanoparticles. The laser-ablated metal nanoparticles have a high purity, because the purity of the metal or metal oxide nanoparticles depends on the purity of target and solvent. The nanoparticles are synthesized in the solution due to the vaporization of targets [20]. Therefore, the metal or metal oxide nanoparticles can be fabricated in the organic and mineral solutions without any agents [20] and contaminants. The laser ablation was used to prepare the metal nanoparticles including Ag–NPs [21], gold (Au–NPs) [22], and copper (Cu–NPs) [23] nanoparticles liquid solution, and laser ablation is also one of the physical methods to prepare the carbon quantum dots. 

As mentioned above, when the laser ablation technique is used to fabricate the nanofluid, the purity of the final product is reliable. Hence, the laser ablation technique is a suitable method for the synthesis of nanoparticles to investigate the action of the optical properties of nanofluid. Moreover, the absorption peak of CD is in the violet [24] range and the wavelength of the laser in the laser ablation technique is usually in the green or near-infrared range of the UV–vis spectrum. Therefore, the laser ablation method can be used for the preparation of pure nanoparticles in the CD solution.

Ag–NPs have a wide range of applications in medicine, biology, and industry. The applications of Ag–NPs to enhancing the Raman shift, as well as the thermal and plasmonic properties, are considerable. Ag–NPs was used to detect the tellurium with covering the graphene hydrogel for enhancement the Raman shift [25] and the detection limit was reported about 100 nM. Ag–NPs were applied to improve the sensitivity of surface-enhanced Raman spectroscopy in the graphene sheet and the enhancement factor was about 6.53 times the pure graphene sheets [26]. To apply the medical diagnostic using Raman spectroscopy, Ag–NPs was considered to enhance the Raman shift for testing the living cancer cells and the toxicity was detected in living cell directly [27]. Hence, Zhang et al. [28] applied the Ag–NPs to detect lung cancer and they could enhance the sensitivity of Raman scattering for analysis of the serum to investigate lung cancer [28]. Ag–NPs can interact with DNA; hence, graphene/Ag–NPs were used to detect the DNA molecules and Ag–NPs have been able to enhance the Raman shift besides interaction with DNA [29]. 

Ag–NPs can interact with carbon-based nanomaterials such as graphene quantum dots, carbon nanotube and carbon quantum dots to enhance the physical and chemical properties of the composite. For example, Ag–NPs was synthesized in commercial graphene quantum dots using laser ablation, and the Raman signal was achieved to be six times better than pure commercial graphene quantum dots [21]. Ag–NPs were synthesized in CD for enhancement of the photocatalyst, Raman scattering, and antibacterial properties, and the results authenticated the interaction of Ag–NPs with CD [30]. Moreover, CD can cap the Ag–NPs for increasing the Raman scattering for sensor application and it has been able for sensing 4-aminothiophenol with detection limit about 5.0 × 10^−11^ M [15]. Bhunia et al. fabricated the CD/Ag–NPs to obtain effective platforms for enhancement of the Raman shift and they prepared the active flexible film to show that the plasmonic field of silver nanoparticles can couple with CD extinction to improve the Raman shift [31]. Lu et al. decorated the reduced graphene oxide by silver and gold nanoparticles to enhance the Raman shift [32]. They could detect the aromatic molecules and DNA with the detection limit about nM [32]. Consequently, Ag–NPs/CD composite has the potential for enhancement of the Raman shift for the detection of the chemical materials. Ag–NPs contains free electron and it has high plasmonic property; hence, it can enhance the fluorescence and Raman properties. Therefore, the Ag–NPs and the metal nanoparticles such as gold and copper are suitable candidates to enhance the optical properties of composite materials and interact with toxic chemicals [33]. 

Fluoride ((C_6_H_4_)_2_CH_2_) is a member of polycyclic aromatic hydrocarbons (PAHs), and it is a toxic organic component. Thus, it causes the pollution of the environment. The analytical methods, including nuclear magnetic resonance (NMR), high-performance liquid chromatography (HPLC), and Fourier transform infrared spectroscopy (FT-IR), were used to investigate the concentration of fluoride.

Raman spectroscopy is a versatile and merited technique to characterize the chemical components, and it can be used to recognize the fingerprint of molecules and chemical components. Raman scattering is based on vibrational modes of molecules and it uses the inelastic scattering of photons [34] when a monochromatic light source interacts with molecules. The cross-section of Raman scattering is small. Hence, the Raman signal is very weak [35] in comparison with other analytical methods such as FTIR signals [36]. Consequently, the enhancement of the Raman signal is a significant activity which helps to obtain the reliability signals for sensor applications [37].

In a previous study, Ag–NPs were prepared in the commercial graphene quantum dots [21] and the Raman shift was investigated without any application. Moreover, the nonlinear refractive index and thermal effusivity were not considered. As mentioned above, Ag–NPs/CD have various physical properties and have the potential to interact with chemical components for sensor application. Fluoride is a toxic chemical and the detection of fluoride is a significant activity in the environment; hence, the interaction of Ag–NPs/CD with fluoride are considered using Raman spectroscopy. In this study, the CD solution was derived from empty fruit bunch biochar (EFBB) using an acid-free hydrothermal method and the Au–NPs was fabricated in prepared CD solution using laser ablation technique in the different ablation time. The main idea is the investigation of optical, thermal properties and detection of fluoride using Raman spectroscopy. The prepared samples were characterized using Fourier transform infrared spectroscopy, transmission electron microscopy, UV-visible, and Raman spectroscopy. The linear, nonlinear optical properties and thermal properties were investigated using UV-visible spectroscopy, z-scan, and photoacoustic techniques, respectively. Then, Ag–NPs/CD was used to detect the fluoride in the aqueous solution using Raman spectroscopy. 

## 2. Results

**Figure 1:**
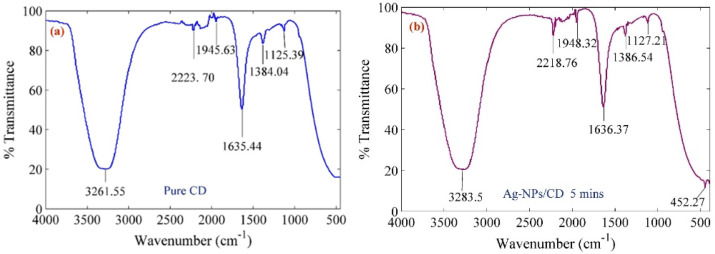
FTIR spectrum of (**a**) pure CD (before ablation the Ag plate) (**b**) Ag–NPs/CD nanocomposite at 5 min ablation time.

Figure 1a,b proves the formation of silver nanoparticles in the CD solution from the FTIR spectrum for pure CD and Ag–NPs/CD composite at 5 min ablation time. The FTIR absorption peaks occurred at 3261.55, 3283.5, 2223.70, 2218.76, 1945.63, 1948.32, 1635.44, 1636.37, 1384.04, 1386.54, 1125.39, 1127.21, and 452.27 cm^−1^. The peak at 452.27 cm^−1^ is in the fingerprint area that assigned Ag–NPs [37,38]. The peaks at 3261.55, 3283.5 cm^−1^ related to the OH group stretching and the peaks at 2223.70 and 2218.76 cm^−1^ assigned the stretching vibrations of the carboxyl groups at the edge and surface of the CD. The peaks at 1945.63 and 1948.32, 1635.44 and 1636.37 cm^−1^ corresponded the stretching vibration of C=O at the edge of the CD molecule structure. The peaks at 1384.04 and 1386.54 cm^−1^ depict the C=C stretching vibration on the benzene ring of CD and another set of peaks appeared at 1125.39 and 1127.21 cm^−1^ assigned the C=C stretching vibration and the asymmetric and symmetric stretching vibrations of the C–O–C groups [39,40]. As a result, the intensity of peaks for the stretching vibration of C=O at the edge of CD molecule structure, the C=C stretching vibration on the benzene ring of CQD, the C=C stretching vibration and the asymmetric and symmetric stretching vibrations of the C–O–C groups changed when the Ag–NPs formed in the CD solution and the peaks at 452.27 cm^−1^ appeared in the FTIR fingerprint area. Consequently, the CD capped the Ag–NPs from the edge of the molecule. 

**Figure 2:**
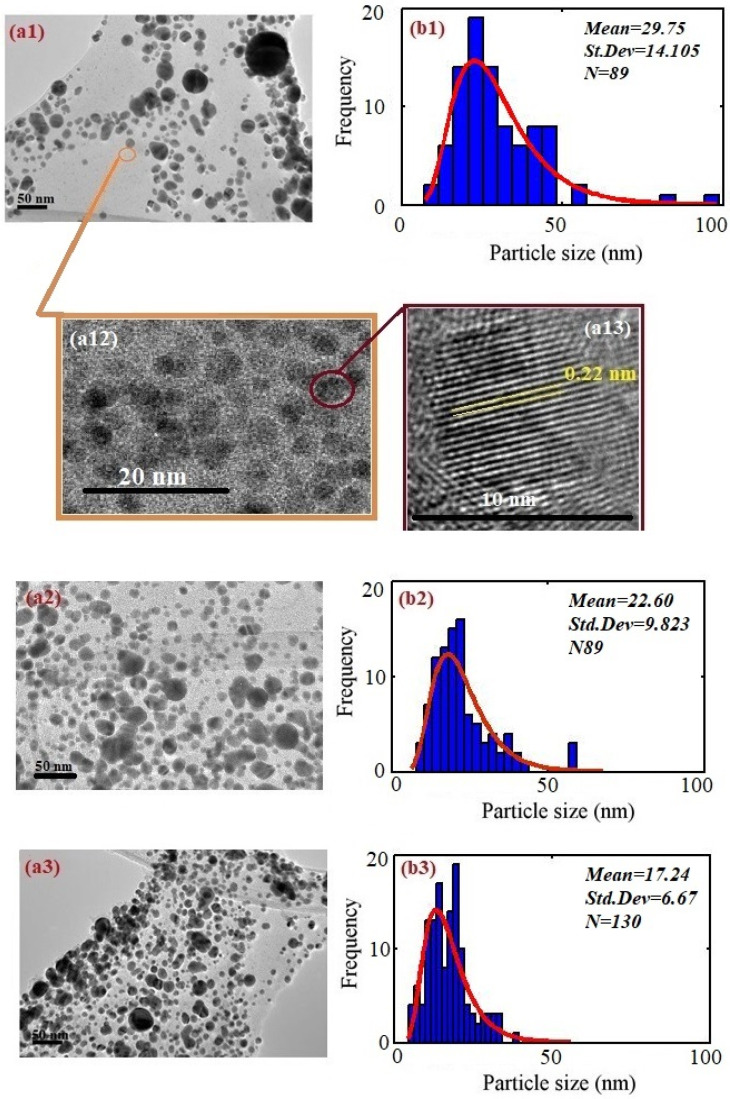
TEM image for Ag–NPs/CD and analysis of image using Image J: (**a1**,**b1**) 5 min; (**a2**,**b2**) 15 min; and (**a3**,**b3**) 20 min. (**a12**) is the high magnification of the orange area and it shows the CD image. (**a13**) is a high magnification of the pink area and the lattice size is about 0.22 nm.

Figure 2 shows the TEM image and analysis of Ag–NPs/CD for 5 min (Figure 2a1,b1), 15 min (Figure 2a2,b2) and 20 min (Figure 2a3,b3). The nanoparticle has a spherical shape and the particle size is within the range from 29.75 nm to 17.24 nm. The TEM images revealed that when the ablation time increases, the particle size decreases. They are authenticated by the blue shift in the UV-visible spectrum. Figure 2a12,a13 shows the CD near the Ag–NPs. The particle size about 4 nm and the high magnification in Figure 2a12 depicts the lattice size of CD is about 0.22 nm [41]. The TEM image proves the Ag–NPs formed in the CD solution. 

**Figure 3:**
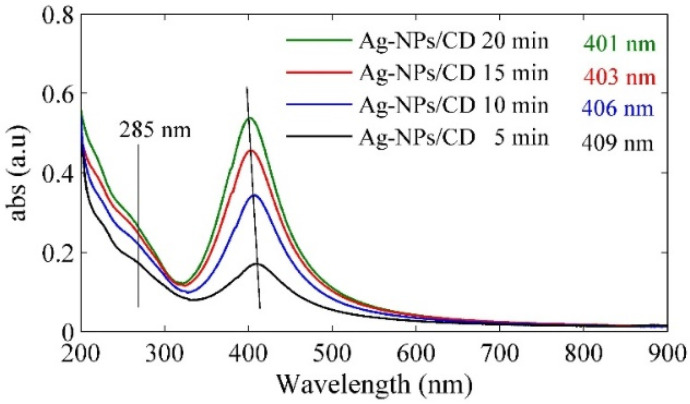
The UV–vis spectra of Ag–NPs/CD in a different ablation time.

Figure 3 shows the UV-visible spectra of Ag–NPs/CD composite solutions. The main peaks appeared at 285 nm, 409 nm, 406 nm, 403 nm, and 401 nm for pure CD and Ag–NPs/CD in 5 min, 10 min, 15 min, and 20 min, respectively. The peaks at 409 nm, 406 nm, 403 nm, and 401 nm were produced from the localized surface plasmon resonance of Ag–NPs. The blue shift is observed in the spectra that authenticated the decrease of particles size as the ablation time increases. This phenomenon can be explained with the Mie theory [42].

The concentration of Ag–NPs was measured using atomic absorption spectroscopy and the values of volume fraction of Ag–NPs were obtained from Equation (1) to determine the volume fraction of Ag–NPs in CD [43] as follows:

(1) $$V=\frac{V_{a}}{V_{a}+V_{b}}=\frac{C_{Ag-NPs}}{C_{Ag-NPs}+\rho }$$

where $C_{Ag-NPs}$, ρ, *V_a_*, and *V_b_* are the concentration of Ag–NPs, the density of Ag–NPs, the volume of Ag–NPs, and the volume of CD solution, respectively. *V_a_* is the ratio of mass and density (*m*/*ρ*) of Ag–NPs [44]. Table 1 shows the pertinent parameters. 

**Figure 4:**
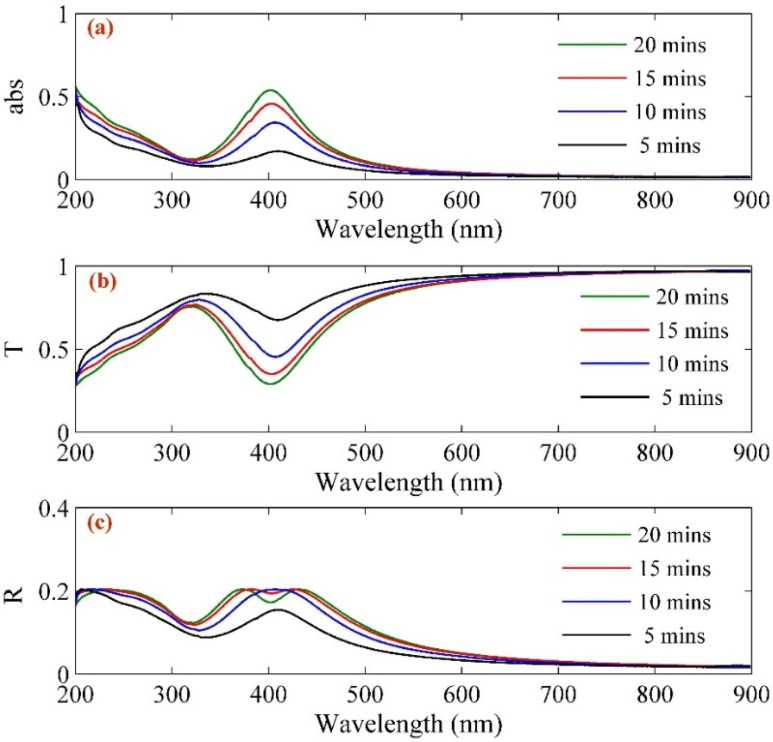
(**a**) absorbance, (**b**) transmissivity, and (**c**) reflectance of Ag–NPs/CD in different ablation time.

Figure 4 shows the results of transmissivity and reflectivity spectra of Ag–NPs/CD composite solution that are within the range from 200 nm to 900 nm. The transmissivity and reflectivity spectra using Beer’s Lambert low provided the optical properties of Ag–NPs/CD composite solution. This study used the absorption and extinction coefficients of the composite to determine the absorption and transmission of the light beam by Ag–NPs/CD composite. Transmissivity (*T*) has a relationship with the absorbance of Ag–NPs/CD composite in different concentrations as follows [45]:

(2) $$absorbance=optical\;density=-\log_{10}^{}T$$

Transmissivity is the ratio of the intensity of the transmitted beam to the intensity of the incident light beam *(I/I*_0_*)*. The absorption coefficient (*α*) and the optical path (*l*) are proportionate to the optical density of Ag–NPs/CD composite as follows [45]:

(3) OD=0.434×α×l

where the optical path was 1 cm and the absorption coefficients for different ablation time increased from 0.0941 to 0.8491. The absorption coefficient and wavelength (*λ*) are directly proportional to the extinction coefficient or imaginary part of the refractive index as follows:

(4) $$k=\frac{\alpha \times \lambda }{4\pi }$$

According to Fresnel theory, the optical properties of a medium such as real and imaginary parts of refractive index depend on reflectivity (*R*) as follows:

(5) $$R=\frac{{\left(n-1\right)}^{2}+k^{2}}{{\left(n+1\right)}^{2}+k^{2}}$$

**Figure 5:**
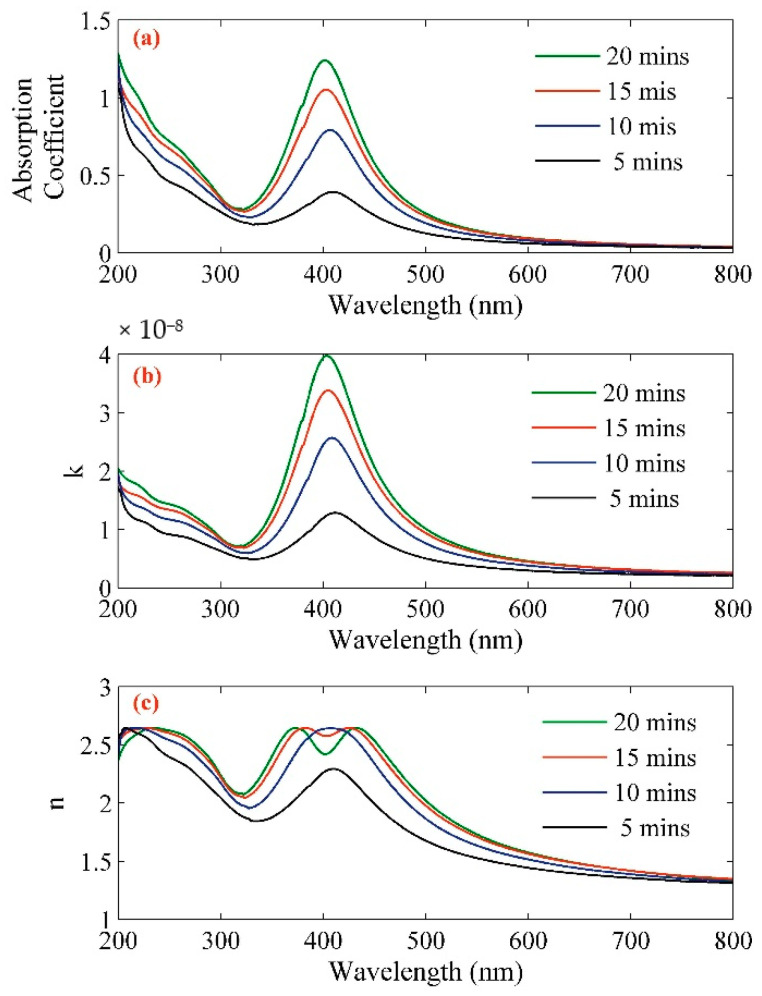
(**a**) Absorption coefficient, (**b**) real part, and (**c**) imaginary part of refractive indices of Ag–NPs/CD composite solution in different ablation time.

Figure 5 shows the variation of the absorption coefficient, the imaginary part of the refractive index, and the real part of the refractive index of Ag–NPs/CD composite. The results were taken from reflectivity spectra and Equation (5) within the range from 200 nm to 900 nm (Figure 4). Table 1 shows the numerical value of real and imaginary parts of the refractive index and absorption coefficient for 532 nm wavelength. 

**Figure 6:**
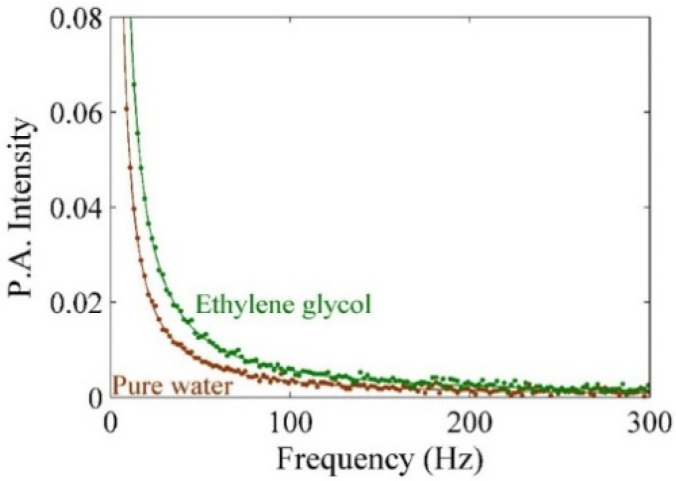
Photoacoustic signals to obtain the setup constant for calibration and test the setup. The thermal effusivity of DDW and pure ethylene glycol are 0.159 W s^1/2^ cm^−2^ K^−1^ and 0.093 W s^1/2^ cm^−2^ K^−1^, respectively.

Figure 6 shows the photoacoustic signal for ethylene glycol. The dotted point represents experimental value, and the solid line is the theoretical fitting curve for the analysis of the experimental value. The amplitude of the signal is a function of the modulation frequency of chopper based on RG theory for explaining photoacoustic signals [46]. The photoacoustic signals that were registered in the presence of Ag–NPs/CD have the information about the amplitude of pressure fluctuations (δP). Hence, δP can be calculated using RG formula as follows:

(6) |δP|=P1fP2(1+P3f+P322f)12

where $P_{1}$, $P_{2}$, and $P_{3}$ are the setup constant which should be obtained by calibrating the setup, and they are functions of chopper frequenc (f) [47]. Hence, aluminium foil 0.0017 cm was used to obtain the setup constant. The $P_{3}$ are a function of, the thermal diffusivity ($\alpha_{Al}$), thermal effusivity ($\varepsilon_{Al}$), and thickness ($l_{Al}$) of the aluminium foil, respectively as follows:

(7) P3=2εsεAllAl(αAlπ)12

It is necessary to have the setup constants so as to determine the thermal effusivity of the CD and Ag–NPs/CD solution. Hence, the setup constants ($P_{1}$ and $P_{2}$) must be obtained by fitting the experimental data to Equation (6) [34] as follows [38]:

(8) $$\left|\delta P_{Al}\right|=P_{1}f^{P_{2}}$$

**Figure 7:**
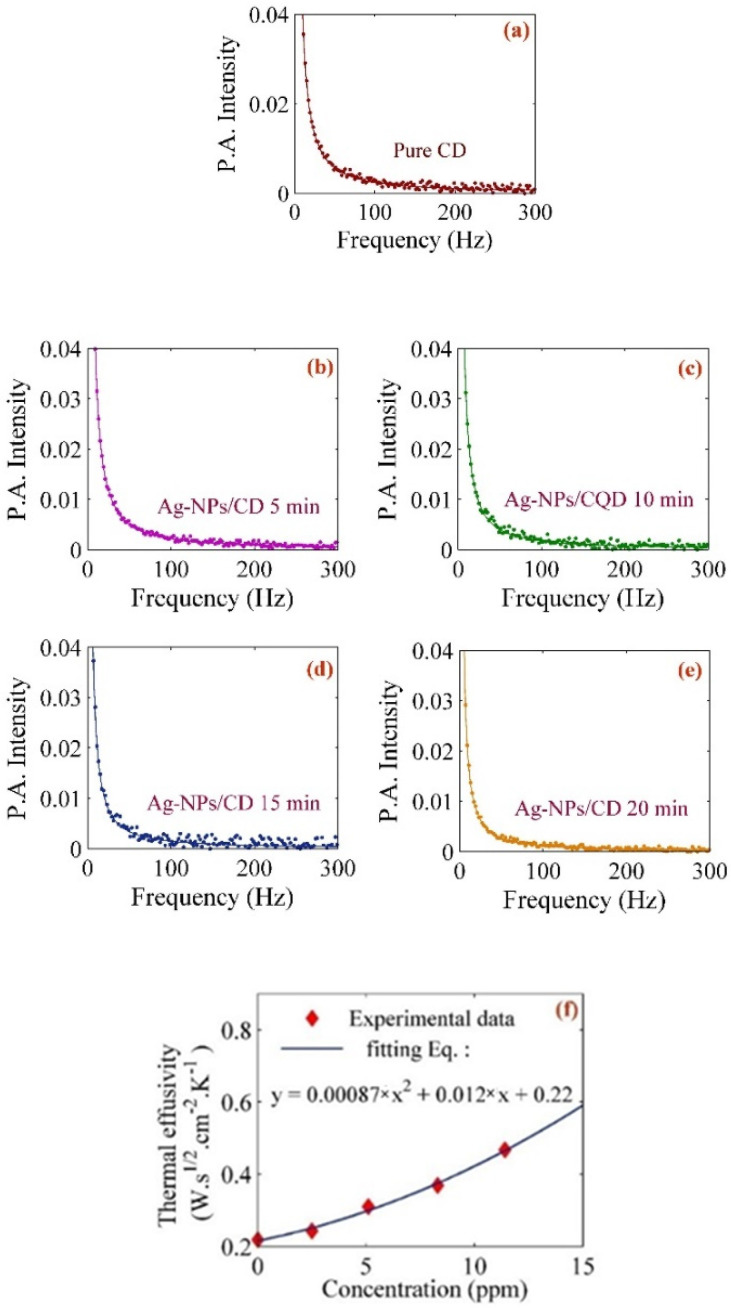
Photoacoustic signals used to determine the thermal effusivity of the (**a**) pure CD, Ag–NPs/CD (**b**) 5 min, (**c**) 10 min, (**d**) 15 min, and (**e**) 20 min ablation times. (**f**) Variation of thermal effusivity for Ag–NPs/CD with concentration.

The experiment performs using pure ethylene glycol and distilled deionized water (DDW) so as to calibrate and obtain the setup constant (see Figure 5). Figure 7a–f depict the photoacoustic signals for the measurement of pure CD, Ag–NPs–CD solution in 5, mins, 10 min, 15 min, and 20 min. Thermal effusivity of pure CD is 0.218 W s^1/2^ cm^−2^ K^−1^ and the thermal effusivity of Ag–NPs/CD in 5 min, 10 min, 15 min, and 20 min ablation times are 0.243 W s^1/2^ cm^−2^ K^−1^, 0.310 W s^1/2^ cm^−2^ K^−1^, 0.369 W s^1/2^ cm^−2^ K^−1^, and 0.467 W s^1/2^ cm^−2^ K^−1^, respectively. The numerical values are listed in Table 2.

The concentration of the pure CD solution was 50 ppm. Figure 6f shows the variation of thermal effusivity for composite solutions with a concentration of Ag–NPs. The thermal effusivity was enhanced by increasing the concentration of Ag–NPs from 0.243 W s^1/2^ cm^−2^ K^−1^ to 0.467 W s^1/2^ cm^−2^ K^−1^. The thermal effusivity is related to heat exchange of Ag–NPs/CD with the surrounding area of the samples. The effective surface expanded by increasing the concentration of Ag–NPs (ablation time) because the distribution of nanoparticles increased, and the particle size of Ag–NPs decreased. Hence, the heat exchangeability of the Ag–NPs/CD increased when the concentration of Ag–NPs (ablation time) also increased. Consequently, the thermal effusivity was enhanced, thereby leading to the improvement of thermal properties of the CD solution. 

**Figure 8:**
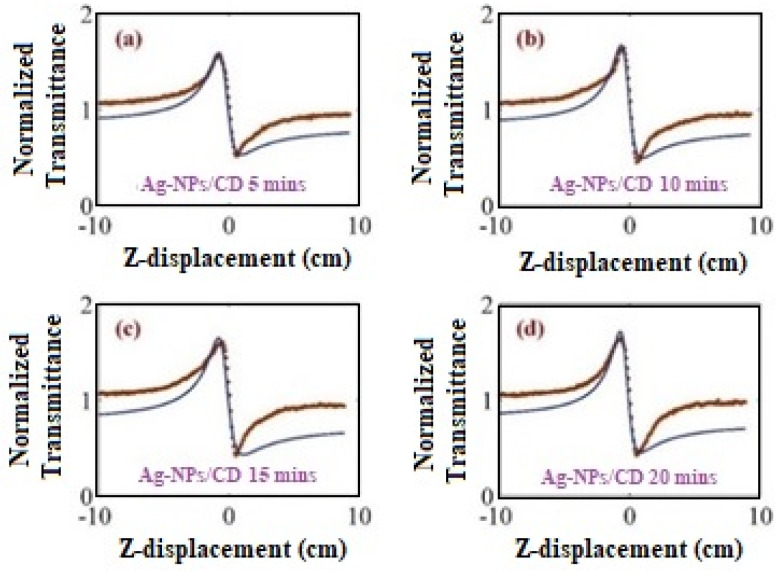
The Z-scan results show the variation of transmittance of the laser beam with the distance for the different ablation time of Ag–NPs/CD solution. (**a**) 5 min, (**b**) 10 min, (**c**) 15 min, and (**d**) 20 min.

The homemade Z-scan setup tested the Ag–NPs/CD composite solution to obtain the nonlinear refractive index (see Figure 13). Figure 8 shows the closed Z-scan signals of Ag–NPs/CD composite solution for different ablation times. The Z-scan signal was fitted with the normalized transmittance from a near-field closed aperture equation as follow:

(9) $$T_{N}^{TLM}={\left(1+\theta \frac{2x}{1+x^{2}}\right)}^{-1},$$

where θ is the on-axis phase shift, and x is the ratio of z-position to Rayleigh length ($x=z/z_{r}$). The on-axis phase shift can be expressed as:

(10) $$\theta =\frac{2\pi Ln_{2}I_{0}}{\lambda \varepsilon_{0}cn_{0}}=\frac{\pi \alpha_{0}\omega_{0}^{2}L}{2\lambda \kappa }\frac{\partial n}{\partial T}I_{0},$$

where L is the sample thickness, $I_{0}$ is the on-axis intensity at the focal point, $\omega_{0}^{}$ is the beam waist of the laser, c is the speed of light, κ is the thermal conductivity, $\frac{\partial n}{\partial T}$ is the thermo-optics coefficient, $\varepsilon_{0}$ is the permittivity of free space and $n_{0}$ is the refractive index of the sample. Therefore, the value of the nonlinear refractive index $n_{2}$ can be found by fitting the equation 1 to the closed Z-scan experimental data. Figure 8 shows the dotted points that are the experimental value of transmittance and the theoretical fitting with Equation (1) are shown as the blue lines. The nonlinear refractive index was within the range from −7.3769 × 10^−10^ to −9.5269 × 10^−10^. Once the nonlinear refractive indices are determined, the real part of nonlinear optical susceptibility can be found from Equation (11) [40,48], and the nonlinear refractive indices and the real part of nonlinear susceptibilities of the samples are listed in Table 3.

(11) Re[x3(esu)]=10−4ε0c2n02πn2(cm2W).

**Figure 9:**
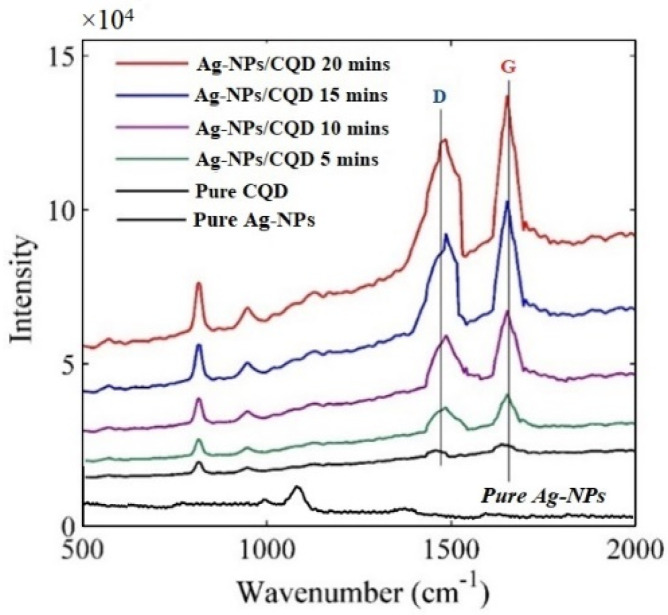
Raman shift spectrum for pure Au–NPs in water at 20 min, a pure CD solution, Ag–NPs/CD 5 min, 10 min, 15 min and 20 min composite solutions.

The Raman specification of nanoscale materials depends on the interaction of light with vibrating of nanomaterials molecule [49]. Figure 9 shows the Raman spectrum of pure Ag–NPs in water at 20 min, pure CD solution and Ag–NPs/CD composite solutions that were excited using the green laser (532 nm). As a result, the peaks at 1014.32 and 1200.23 cm^−1^ corresponded to Au–NPs and the peaks are very week. The D and G peaks appeared in the spectrum that corresponded to aromatics domains carbon quantum dots. The peak at 1456.31 cm^−1^ is linked to sp^3^ orbital hybridized of C=C (carbon to carbon bonds), C=O (carbon to oxygen bonds), and COOH (hydroxyl group), respectively. The peaks at 1638.63 cm^−1^ corresponded to C–C as a result of sp^2^ carbon orbital. The Raman spectra show that Ag–NPs can enhance the peak of Raman intensity, and the scattered Raman intensity of Ag–NPs/CD solution at 5, 10 15, and 20 min which were 1.8, 2.4, 3.2 and 4.8 times greater than scattered Raman intensity of pure CD. The concentration of Ag–NPs causes to enhance the intensity of Raman scattering. As mentioned above, Ag–NPs improved the thermal effusivity of the composite. Consequently, the phonon vibration enhanced. Raman eventuates the interaction of light with molecular vibration. On the other hand, Raman signals obtained from the interaction of a photon with phonon. CD and Ag–NPs interact together and Ag–NPs were capped with a functional group of CD which prevents the aggregation of the Ag–NPs. Ag–NPs can enhance the phonon vibration; hence, the Raman intensity or Raman properties have been enhanced. Figure 9 shows the intensity peaks for CD solution and Ag–NPs/CD composite solutions. The ratio area under D peak and G peaks increased when the concentration of Ag–NPs increases that confirmed the increase of scattered Raman intensity when the concentration of Ag–NPs is increased [50].

The photons in the visible and infrared wavelength can produce the Raman effect. The photons pass through the sample and create an oscillating polarization in the molecular structure. The combination of the oscillating polarization of molecule couple and the vibrational mode of the molecule can change the vibrational state that scatters the photon. In the visible range, the silver nanoparticles have a strong plasmonic effect. Hence, the excitation of localized surface plasmon can enhance the intensity of scattering Raman [35,36].

**Figure 10:**
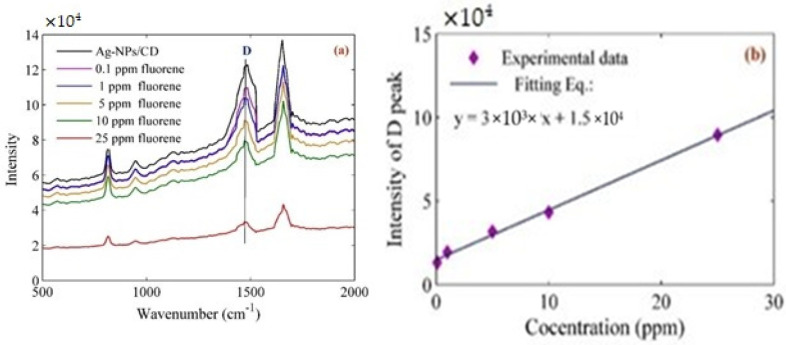
(**a**) Raman spectrum for the detection of low concentration (0.1, 1, 5 10, and 25 ppm) of fluoride; (**b**) variation of D band intensity with the concentration of fluoride.

The fluoride in 0.1, 1, 5, 10, and 25 ppm concentrations was mixed separately with Ag–NPs/CD ×10^4^ composite. Figure 10a shows the Raman spectrum when the Ag–NPs/CD composite contacted with the fluoride. The Raman peak decreased when the concentration of fluoride increased. Figure 10b shows the variation of intensity peaks with the concentration of fluoride. The Ag–NPs/CD composite can interact with fluorideand it is sensitive to the low concentration of fluoride. The Raman test is conducted repeatedly for five times to estimate the stability of the Raman properties and Raman spectrum of the Ag–NPs/CD composite solution in the presence of fluoride. The average intensity shift and the standard deviation (σ) were 1.346 × 10^4^ and 4.84 × 10^2^, respectively. The limit of detection (LOD = 3σ/K) is proportional to standard deviation and slope (K) of the variation of Raman intensity with the concentration of fluoride. Therefore, the LOD was about 66.03. 

The Ag–NPs can enhance the nonlinear and Raman properties of CD. The volume fraction increases when the ablation time and concentration of Ag–NPs increased. The increase of scattering cross-section had caused the increase of optical nonlinear and Raman properties of CD.

**Figure 11:**
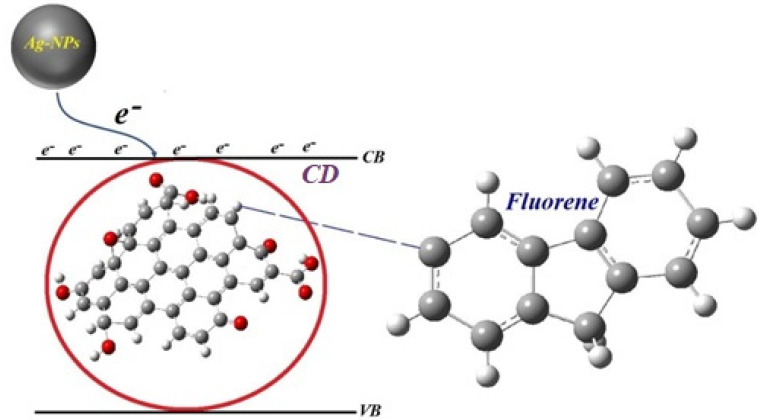
The diagram for the integration of Ag–NPs/CD with fluoride.

The main mechanism (see Figure 11) of the Raman sensor for the detection fluoride depends on transfer electron between Ag–NPs and CD because Ag–NPs contains a free electron and have a high plasmonic property. Thus, Ag–NPs/CD can interact with the toxic chemical and it is a suitable platform for the detection of fluoride. This mechanism could explain based on surface-related defective sites. It generally refers to any sites that have nonperfect sp^2^ domains, which result in surface energy traps. Both sp^2^ and sp^3^ hybridized carbons, and the carbonyl and carboxylic groups as a localized electronic state, which contribute to the CD and Ag–NPs/CD Raman shift. 

## 3. Materials and Methods 

### 3.1. Reagents 

Biochar from an empty fruit bunch (EFBB), fluoride, isopropanol (assay 98%), and high purity silver plate was provided from Parka Go Green SenBud, Merck Company (Darmstadt, Germany), and Sigma Aldrich (St. Louis, MO, USA), respectively. The solvents were of analytical grade. 

### 3.2. Preparation of Carbon Dots

Ref. [51] reported the preparation of CD. Briefly, the CD was prepared from empty fruit bunch biochar (EFBB) in acid-free synthesis. The carbon source came from EFBB, while the co-solvent was isopropanol in a 3:1 ratio water/isopropanol mixture. The process added 0.06 g of EFB biochar to a steel tube. Then, the same tube was added with 6 mL of the co-solvent mixture before it is sonicated for 5 min before it is transferred into an oven with a temperature of 250 °C for 60 min. After completing the heating process, the steel tube was submerged in the water bath for about 4 h. It produced a black liquid that was purified using centrifugation (4000 rpm, 5 min) to remove residual biochar [52,53,54,55]. The supernatant containing CD was obtained and kept for further classification. 

### 3.3. Laser Ablation 

**Figure 12:**
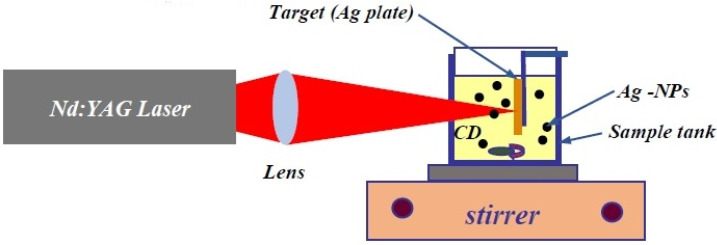
The preparation of Ag–NPs in the CD solution using laser ablation setup. The set up contains a high-power laser, a lens, a stirrer, a sample tank, and a target.

The silver nanoparticles (Ag–NPs) were prepared using the laser ablation methods with Nd: YAG (1064 nm). The setup (Figure 12) contains a laser, stirrer, lens, sample tank, and silver plate (purity 99%) [56]. The energy of the laser beam and the repetition rate were 900 mJ and 40 Hz, respectively. As a literature, the minimum energy for the ablation of metal nanoparticles such as silver and gold is about 650 mJ [20]. The silver plate was immersed in 50 ppm concentration of CD solution and the laser ablation times were 5 min, 10 min, 15 min, and 20 min for preparation of Ag–NPs. UV-visible spectroscopy (Perkin Elmer, Waltham, MA, USA), transmission electron microscopy (TEM, Jeol, Tokyo, Japan), Fourier transform spectroscopy (FT-IR, Thermo Fisher Scientific, Waltham, MA, USA), and Raman spectroscopy was used to characterize the prepared samples. (to control the Raman result, the Ag–NPs was also prepared during 20 min ablation time in the water.) 

### 3.4. Z-Scan Setup

**Figure 13:**
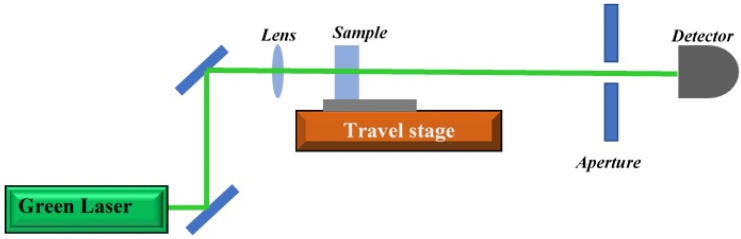
Z-scan setup contains a high-power laser, a traveling stage, an aperture, a mirror, a lens, and a detector. The nonlinear refractive indices of CD and Ag–NPs/CD were measured using high power laser at the wavelength of 532 nm.

Figure 13 shows the home-made z-scan setup for measurement the nonlinear refractive index. The high-power laser was used to do the experiment. The laser was a continuous-wave diode-pumped solid-state laser (Coherent Verdi-V5, Coherent, Santa Clara, CA, USA), and it was operated at the wavelength of 532 nm. The high-power stability of 1% and the pointing stability of less than 2 µrad/°C of the laser are favorable for producing accurate z-scans. The sample was placed in a 1 mm path length quartz cuvette where the path length is smaller than the laser’s Rayleigh length of 3.12 mm. Therefore, this experimental condition fulfils the precondition for the z-scan analysis of a thin sample. The sample in the cuvette was positioned on the Thorlabs’s LTS-300 (Thorlabs, Newton, MA, USA) computerized linear stage, where the translation axis is aligned parallel to the beam of the laser. The laser beam was focused on the sample with a positive lens with 20 cm focal length. During the z-scan experiment, the cuvette was moved from −100 mm position to +100 mm position parallel to the laser beam direction. For reference, the negative position distance was measured from the middle of the cuvette to a point nearer to the focusing lens. A photodiode was used to record the transmitted laser beam through the sample. The photodiode was placed after an aperture to capture the closed z-scan data [46,57,58]. 

### 3.5. Photoacoustic Setup 

**Figure 14:**
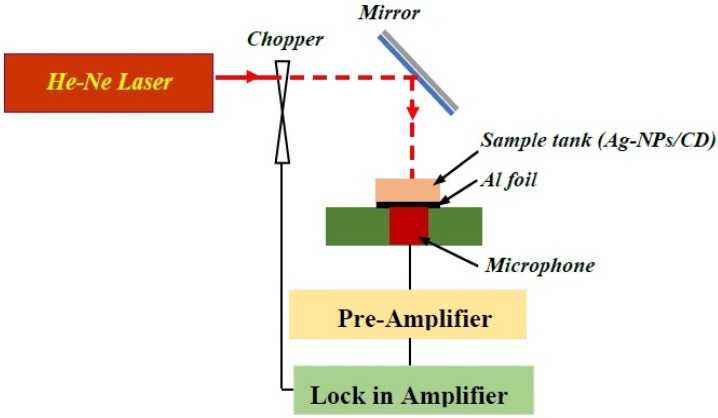
The photoacoustic setup contains a He–Ne laser, a chopper, mirror, sample tank, Al foil, microphone, pre-amplifier and lock-in amplifier.

Figure 14 shows the photoacoustic setup [38,47] for the measurement of the thermal effusivity of CD and Ag–NPs/CD. The setup works based on the interaction of a He–Ne laser with sample and generation of phonon. The main components of the photoacoustic setup are the He-Ne laser (75 mW, 632.8 nm), electret microphone and a sample tank. The experiments were carried out at room temperature and the He–Ne laser was modulated with a chopper, and the laser beam interacted with the sample after reflection from the mirror. The signal was amplified using a preamplifier and lock-in amplifier. During the experiment, the chopper frequency was shifted from 21 Hz to 300 Hz, and it was controlled by a special computer program.

The bottom of the sample tank was closed and shut using a 0.0017 cm thick aluminium sheet, and the sample tank was filled with the CD or Ag–NPs/CD. The amplitude and phase of photoacoustic signals were registered using the microphone, and they were analyzed based on Rosencwaig–Gersho (RG) theory to determine the thermal effusivity of the Ag–NPs/CD composite solution. 

### 3.6. Raman Spectroscopy

The Raman spectrum was registered using confocal Raman spectrometer (alpha300 R, WITec, Ulm, Germany,) at room temperature and the excitation wavelength was 532 nm. The spectrum was analyzed using a blaze grating with 600 g/mm and BLZ 500 nm. The width and height resolutions were 1024 and 127 pixels, respectively. 

### 3.7. Preparation of Fluoride Solution

To prepare the fluoride solutions, 1 g of pure fluoride was dissolved in 1000 cc of pure hexane to obtain the 1000 ppm concentration of fluoride. The prepared solution was systematically dissolved in a solvent to achieve the 0.1, 1, 5, 10, and 25 ppm concentrations of fluoride. 

## 4. Conclusions

The Ag–NPs/CD composite aqueous solutions were prepared using a laser ablation technique in CD solution. The ablation times were 5, 10, 15, and 20 min. The prepared samples were tested using the analytical method and from the FTIR, UV–vis, and TEM images, Ag–NPs were formed in the CD solution in the spherical shape with particle size in the range of 29.75 nm to 17.24 nm. The nonlinear refractive index and real part of nonlinear susceptibility were in the range of 7.3769 × 10^−10^ to 9.5269 × 10^−10^ cm^2^ W^−1^ and 5.46 × 10^−8^ to 6.97 × 10^−8^ esu and the thermal effusivity increased from 0.243 W s^1/2^ cm^−2^ K^−^^1^ to 0.467 W s^1/2^ cm^−2^ K^−1^. Therefore, when the ablation time increased from 5 min to 20 min, the particle size decreased, but the thermal effusivity, nonlinear refractive index, and nonlinear susceptibility increased. The Raman intensity increased when the concentration of Ag–NPs increased from 2.5 ppm to 11.4 ppm and the Ag–NPs/CD used to detect the fluoride in low concentration and the detection limit was about 0.1 ppm.

## Figures and Tables

**Table 1:** Pertinent parameters of Ag–NPs/CD composite solution.

Sample	Concentration (ppm)	Volume Fraction (×10^−6^)	Absorption Coefficient (at 532 nm)	*n* (at 532 nm)	*k* × 10^−9^ (at 532 nm)
Ag–NPs/CD 5 min	2.5	0.130	0.094	1.5676	3.94
Ag–NPs/CD 10 min	5.1	0.264	0.134	1.6979	5.65
Ag–NPs/CD 15 min	8.3	0.430	0.163	1.7836	6.87
Ag–NPs/CD 20 min	11.4	0.591	0.173	1.8129	7.32

**Table 2:** The thermal effusivity of the adjustment parameter (*P_3_*) of Ag–NPs/CD composite solutions.

Sample	*P_3_* (×10^2^)	Thermal Effusivity (W s^1/2^ cm^−2^ K^−1^)
Water	0.469	0.159
Ethylene Glycol	0.274	0.093
Pure CD	0.631	0.218
Ag–NPs/CD 5 min	0.717	0.243
Ag–NPs/CD 10 min	0.914	0.310
Ag–NPs/CD 15 min	1.088	0.369
Ag–NPs/CD 20 min	1.377	0.467

**Table 3:** The nonlinear refractive index and real part of nonlinear optical susceptibility.

Samples	−*n*_2_ (×10^−10^ cm^2^ W^−1^)	−*R_e_* (x^3^ × 10^−8^ esu)
Ag–NPs/CD 5 min	7.377	5.40
Ag–NPs/CD 10 min	9.556	6.99
Ag–NPs/CD 15 min	9.184	6.71
Ag–NPs/CD 20 min	9.527	6.97
